
JOURNAL INFORMATION
==============================
NLM Title Abbreviation: Sci China Life Sci
Journal ID: Sci China Life Sci
NLM Journal ID: 101529880

Science China. Life Sciences

ISSN: 1674-7305
EISSN: 1869-1889

ARTICLE INFORMATION
==============================
PMCID: PMC7088720
PMID: 24526493
DOI: 10.1007/s11427-014-4624-3
Article ID: 4624
Article version: 1
Subjects: Insight

Thinking from event of Kangtai hepatitis B vaccination

Ruan Li ruanl@bbn.cn

grid.198530.6 0000000088032373 Biotech Center for Viral Disease Emergency, Institute for Viral Disease Control and Prevention, Chinese Center for Disease Control and Prevention, Beijing, 102206 China
Electronic publication date: 2014 Feb 13
Print publication date: 2014
Volume: 57
Issue: 5
First page: 555
Last page: 556
Received 2013 Dec 26; Accepted 2014 Jan 31
Copyright: © The Author(s) 2014
License: Open AccessThis article is distributed under the terms of the Creative Commons Attribution 4.0 International License (https://creativecommons.org/licenses/by/4.0), which permits use, duplication, adaptation, distribution, and reproduction in any medium or format, as long as you give appropriate credit to the original author(s) and the source, provide a link to the Creative Commons license, and indicate if changes were made.
License URL: https://creativecommons.org/licenses/by/4.0/

Keywords: Carrier Rate, Preventative Vaccination, Disease Emergency, Monkeypox, Viral Disease Control
issue-copyright-statement: © Science in China Press and Springer-Verlag GmbH 2014

==============================
This article is published with open access at link.springer.com

Biographical Sketch

As a doctor and researcher, Ruan Li once held the post of director of Institute for Viral Disease Control and Prevention of Chinese Centre for Disease Control and Prevention and chief of Biotech Center for Virus Disease Emergency. Since 1980, he has focused on the study of heritable variation and immunization of viruses and engaged in the research and development of biological technology including genetically engineered vaccines. In recent years, he was responsible for the monitoring of SARS and prevention and control of poxvirus related diseases such as smallpox and monkeypox. He was also responsible for over 20 research projects sponsored by national science and technology projects, national high-tech research and development plan, major sci-tech project, natural science fund, American NIH, EU and Ministry of Health, amongst others. He established two genetic expression systems using mammalian cells and recombinant vaccinia virus Tiantan strain and successfully researched Hepatitis B genetic engineering vaccine and Phase I clinic trails of five vaccinia recombinant vaccines. He was awarded a certificate of distinguished visiting scientist at Mount Sinai School of Medicine, New York in 1986, a certificate of excellent returned overseas researcher who made outstanding contributions to the state in 1991, a government special allowance in 1991, the National Science Fund for Distinguished Young Scholars (NSFDYS) in 1995, the title of National Expert with Remarkable Contributions in 1996, the first prize of the National Science and Technology Progress in Recombinant Hepatitis B Vaccine Research in 1993 and the second prize of the National Science and Technology Progress in Research and Application of High-Efficiency Expression System Derived from Vaccinia Virus Tiantan Strain. He has also won other four provincial and ministry prizes, was involved in producing over 20 books, applied for 11 patents and published over 200 papers.


References

1 Liu CB Liu XG Qi ZB Xiong SS Epidemiology of viral hepatitis B The Laboratory Diagnosis of Viral Hepatitis 1999 2nd ed. Beijing People Health Publication 107 110
2 Bureau of Disease Control and Prevention of Ministry of Health of China and Chinese Center for Disease Control and Prevention. Report of Seroepidemiology Survey of Viral Hepatitis B in China. Beijing: People Health Publication, 2011
3 Ruan L Zhao K Genetic engineering subunit vaccine Vaccine 2013 Beijing People Health Publication 396 400
4 China Food and Drug Administration and National Health and Family Planning Commission of the People’s Republic of China. Media Briefing on the Event of Hepatitis B Vaccination. http://www.sfda.gov.cn/WS01/CL0051/95394.html, 24 Dec 2013
